# Treatment of Older Adult Patients with Glioblastoma: Moving towards the Inclusion of a Comprehensive Geriatric Assessment for Guiding Management

**DOI:** 10.3390/curroncol29010032

**Published:** 2022-01-14

**Authors:** Manik Chahal, Brian Thiessen, Caroline Mariano

**Affiliations:** Division of Medical Oncology, British Columbia Cancer-Vancouver Cancer Centre, Vancouver, BC V5Z 4E6, Canada; bthiesse@bccancer.bc.ca (B.T.); cmariano@bccancer.bc.ca (C.M.)

**Keywords:** glioblastoma, comprehensive geriatric assessment, elderly patients, management

## Abstract

Glioblastoma (GBM) is the most common primary malignant brain tumor in adults, and over half of patients with newly diagnosed GBM are over the age of 65. Management of glioblastoma in older patients includes maximal safe resection followed by either radiation, chemotherapy, or combined modality treatment. Despite recent advances in the treatment of older patients with GBM, survival is still only approximately 9 months compared to approximately 15 months for the general adult population, suggesting that further research is required to optimize management in the older population. The Comprehensive Geriatric Assessment (CGA) has been shown to have a prognostic and predictive role in the management of older patients with other cancers, and domains of the CGA have demonstrated an association with outcomes in GBM in retrospective studies. Furthermore, the CGA and other geriatric assessment tools are now starting to be prospectively investigated in older GBM populations. This review aims to outline current treatment strategies for older patients with GBM, explore the rationale for inclusion of geriatric assessment in GBM management, and highlight recent data investigating its implementation into practice.

## 1. Introduction

Glioblastoma (GBM) is the most common and most lethal primary brain tumor in adults. Over half of patients with newly diagnosed GBM are over age 65 [1,2] with a peak incidence between 75 and 84 years [3], and as the global population ages, incidence rates continue to increase [4]. Despite standard of care with surgery, radiation, and chemotherapy, prognosis still remains poor with median overall survival (OS) of 15 months [5] and only approximately 9 months for older patients [6].

Age and performance status have consistently been shown to be negative prognostic factors [7], and clearly influence treatment decisions. Furthermore, other factors such as tumor biology, comorbid conditions, polypharmacy, reduced treatment tolerance, and socioeconomic status may additionally influence prognosis for older cancer patients. Older adults with GBM face additional complexities marked by progressive neurologic deficits and neurocognitive decline from the disease and its treatment that can impact self-care and decision-making [8,9]. Of note, the definition of “older adult” in this population is not well established, as unlike for other solid tumors, prospective trials for GBM have used cut-offs ranging from as low as age 60 to 70 to define their older population. Notably, the trial that established the standard of care for GBM management excluded patients over age 70 [5], and consequently extrapolating data from this and other prospective trials to the older adult population becomes problematic. Although chronologic age is becoming less validated as a marker of treatment tolerability, patients over age 65 are typically categorized as older adults, and their treatment considerations are unique relative to the general adult population [10]. Given these considerations and the paucity of randomized clinical trials involving older GBM patients until relatively recently, there has been uncertainty regarding optimal treatment such that nearly 30% of patients over age 65 receive no treatment or less than standard of care [11,12].

As it became apparent that treatment regimens and algorithms for adults with cancer do not necessarily translate to the older adult population, the Comprehensive Geriatric Assessment (CGA) was introduced as a tool in oncology approximately 20 years ago [13]. Since then, several studies have demonstrated a prognostic and predictive role of the CGA in older patients with cancer [14,15]. The use of the CGA in cancer patients has been advocated for by the International Society of Geriatric Oncology (SIOG) [16], and it has been incorporated into the American Society of Clinical Oncology (ASCO) guidelines on the assessment and management of the vulnerable elderly [17]. The use of the CGA and other geriatric assessment tools is starting to be investigated for older patients with GBM. We will review current treatment strategies for older patients with newly diagnosed GBM and explore the rationale for incorporating geriatric assessment into GBM management, along with recent data looking at its implementation.

## 2. Current Glioblastoma Management in Older Adults

### 2.1. Surgery

Due to concerns regarding tolerance because of age, comorbidities, or risk of postoperative complications such as delirium, older GBM patients are often treated with less aggressive surgical management such as biopsy or palliative care [18]. However, a large meta-analysis by Almenawer et al. [19] of high-grade glioma patients over age 60 suggested improved median overall survival for patients receiving gross total resection or partial resection compared to those who only underwent biopsy or best supportive care. They additionally compared Karnofsky performance status (KPS) scales before and after surgery and showed improvement in functional status for older patients receiving resection, while it remained stable or deteriorated after biopsy only. Further supporting the role of maximal resection, a large population-based analysis using the Surveillance, Epidemiology, and End Results (SEER) database in the United States showed that while the frequency that gross total resection is achieved decreases as a function of age, gross total resection conferred a 2 to 3-month overall survival benefit over partial resection in all age groups. This remained true after multivariate analysis adjusted for other patient variables, tumor location, and delivery of radiotherapy, with absolute benefit decreasing with advancing age [20]. There has been only one prospective randomized trial comparing maximal safe resection to biopsy, which was terminated early as outcomes clearly showed a benefit of maximal resection with median survival of 5.6 months vs. 2.8 months [21].

Age and extent of resection are considered two of the strongest prognostic factors in GBM [22], but interpretation of observational data are limited as they do not account for the factors that influence decisions to pursue surgery, which in themselves are often associated with survival [23]. It is unlikely that aggressive surgical management would be suitable for all older patients, and there are no clear objective guidelines to determine which GBM patients are most likely to derive benefit. Frailty, defined as a syndrome of physiological decline in late life characterized by marked vulnerability to adverse health outcomes, has been used to estimate overall health status in geriatric patients [24]. Currently, the majority of surgical treatment decisions and frailty assessments of older patients with GBM are based on age, functional status, and general provider impression as opposed to more objective measures [25,26]. Frailty indexes have been useful for predicting postoperative complications in other cancer surgeries [27,28]. Using the Canadian Study on Health and Aging Modified Frailty Index (mFI) Cloney et al. [29] conducted a retrospective analysis of 319 patients aged 65 or older with pathologically confirmed glioblastoma and assessed surgical outcomes. They showed that frailer patients and those with higher cardiovascular risk were less likely to undergo maximal safe resection and were less likely to undergo re-operation for recurrent disease on multiple regression analysis. Additionally, as frailty score increased, patients had increased overall rate of postoperative complications. Furthermore, frailty was associated with decreased OS independent of age and KPS, as head-to-head comparison of the frailest group with the least frail group demonstrated a median survival difference of 4.7 months. This study therefore suggests a role for validated and objective tools to assess frailty in older GBM patients undergoing craniotomy, as well as rationale for prospective studies evaluating geriatric assessment prior to surgery for GBM.

### 2.2. Radiotherapy

A meta-analysis published in 1979 established the standard of care radiotherapy (RT) dose of up to 60 Gy in 30 fractions for the treatment of GBM in adults [30]. Subsequent data suggest that older patients tolerate standard doses relatively poorly, with higher frequency of fatigue, difficulties complying with the 6-week course, and higher frequency of cognitive defects than their younger counterparts [8,31]. A landmark trial by Roa et al. [32] compared standard RT of 60 Gy in 30 fractions to a hypofractionated course of 40 Gy in 15 fractions in GBM patients over age 60, and found similar OS of 5.1 months vs. 5.6 months, respectively. Additionally, the Nordic GBM trial of patients over age 60 deemed not fit enough for standard chemoradiotherapy confirmed longer survival in patients treated with a hypofractionated regimen of 34 Gy in 10 fractions compared to those receiving standard therapy, with median OS of 7.5 months vs. 6.0 months, respectively, though this was not significant. However, in patients over age 70, survival was significantly improved with hypofractionated RT compared to standard RT [33]. These studies additionally showed that patients treated with the standard RT course required increased corticosteroid dosage and duration relative to those treated with hypofractionated RT. A subsequent randomized control trial showed that an even shorter course of RT of 25 Gy in 5 fractions was noninferior to a hypofractionated course [34]. Although quality of life measures one and two months after treatment were comparable, due to concerns of high radiation toxicity with a single dose of 5 Gy this approach is less widely adopted [8].

The potential for radiation-induced neurologic toxicity and deterioration of quality of life is the main concern for older GBM patients receiving RT. Treatment interruptions due to acute toxicity have been reported in 20 to 25% of patients receiving standard RT compared to <10% receiving a hypofractionated course [32,35]. While quality of life evaluations in prior studies were not powered to make meaningful comparisons, in general older GBM patients treated with hypofractionated RT had stabilization or improvement in quality-of-life scores [35,36]. Although radiation is considered an essential component of GBM management that improves survival without compromising quality of life compared to best supportive care [37], there are very few clinical data on which to base treatment decisions, and the optimal regimen in older patients remains to be established.

### 2.3. Chemotherapy

Alkylating agents have shown the greatest efficacy towards GBM. Temozolomide is a generally well tolerated oral chemotherapeutic agent relative to other alkylating agents such as lomustine, which has more delayed and cumulative hematologic side effects that limit its use in an older, more vulnerable population [8,38]. Although temozolomide is the standard of care chemotherapy for patients with GBM, its efficacy can be limited depending on the molecular profile of the tumor. Mutations in isocitrate dehydrogenase (IDH) and methylation the O^6^-methylguanine-DNA-methyltransferase (MGMT) gene promoter are important prognostic factors in GBM. MGMT methylation specifically is predictive of response to temozolomide [39], as the functional MGMT DNA repair protein is able to repair mutagenic lesions caused by alkylating agents [40]. Mutations in IDH and the glioma CpG island methylator phenotype are much less common in older GBM populations [41], but despite age-associated decrease in general methylation levels in the brain, MGMT promoter methylation is found in nearly half of all GBM diagnosed in older adults [33,42].

Two randomized trials investigated the use of single agent temozolomide for treatment of older patients with GBM. In addition to comparing two radiotherapy schedules in GBM patients over age 60, the Nordic trial incorporated a third arm of temozolomide (200 mg/m^2^ for 5 days with cycles repeated every 28 days for up to 6 cycles). Median OS was significantly longer for patients treated with temozolomide alone compared to standard RT (8.4 vs. 6 months, respectively), and this was further confirmed in patients over age 70. Survival for patients treated with temozolomide compared to hypofractionated RT were comparable, and treatment with temozolomide was associated with significantly longer survival in patients with MGMT promoter methylation compared to those without (9.7 vs. 6.8 months, respectively) [33]. Similarly, the Methusalem (NOA-08) trial conducted in patients aged 65 or older investigated single agent temozolomide (administered at a dose of 100 mg/m^2^ daily one week on, one week off) compared to standard RT of 60 Gy in 30 fractions, and OS showed noninferiority of temozolomide (8.6 vs. 9.6 months, respectively). MGMT methylation did confer increased survival in patients treated with temozolomide, with a median OS of 11.9 months compared to 8.2 months in those without methylation [43]. In a recent update of the NOA-08 trial after median follow up of 7.5 years, the authors confirmed that patients with methylated MGMT had markedly prolonged OS when treated with temozolomide relative to RT (18.4 vs. 9.6 months, respectively) [44]. Therefore, single agent temozolomide appears to be a reasonable treatment for older patients with GBM, especially those with methylated MGMT.

### 2.4. Chemoradiotherapy

Combined modality treatment with 60 Gy RT over 30 fractions and concurrent temozolomide, followed by 6 cycles of adjuvant temozolomide remains the standard of care for adults with GBM as established by Stupp et al. [5]. However, this trial excluded patients over age 70, and only 83 of 573 patients were over the age of 65 [45]. Subsequent age dependent analysis showed that the benefit of the standard of care regimen declines continuously with age, and becomes more closely balanced with risks of toxicity [46]. Therefore, extrapolation of this regimen to older adults is questionable. Given that a hypofractionated RT schedule had become more widely adopted for older patients, in the CCTG CE.6/EORTC 26,062 trial Perry et al., compared hypofractionated RT of 40 Gy in 15 fractions alone to the same RT regimen with concurrent temozolomide (75 mg/m^2^ daily) followed by up to 12 cycles of adjuvant temozolomide in GBM patients aged 65 or older with a KPS of at least 70%. Median OS was improved with combination therapy relative to RT alone (9.3 vs. 7.6 months). Furthermore, not only was the benefit substantial for those with MGMT methylated tumors (mOS 13.5 vs. 7.7 months), but the addition of temozolomide also improved survival in patients with MGMT unmethylated tumors compared to RT alone (10.0 vs. 7.9 months) [6]. More recently, a meta-analysis of 7 randomized control trials identified hypofractionated RT with concurrent and adjuvant temozolomide as the best adjuvant treatment option for older patients with GBM [47]. A summary of pivotal trials informing adjuvant treatment of older adults with GBM is presented in Table 1.

Despite favorable outcomes with temozolomide in the older GBM population, treatment with chemotherapy is not benign. Hurria et al. [48] previously showed that patients over age 65 receiving chemotherapy for any cancer type have a higher prevalence of Grade 3 or higher toxicity. Though this study did not explicitly include CNS tumors, other investigations have similarly shown that side effects of fatigue and hematologic toxicity associated with temozolomide can be more frequent and more severe in older adults [8]. Amongst those enrolled in the EORTC 26,062 trial, patients treated with combination chemoRT suffered increased rates of hematologic toxicity [6], and when compared with younger populations, older adults are at an increased risk of Grade 3 or 4 hematologic toxicity when treated with temozolomide, with rates up to 20 to 30% [6,7,49]. Additionally, prior studies have raised concern of increased neurotoxicity with concurrent temozolomide and radiation, as well as mental status deterioration during adjuvant temozolomide that did not correlate with disease progression in most cases [49,50]. In the real-world setting, the incidence of treatment-related toxicities is much higher than that reported in prospective clinical trials, likely due to presence of comorbidities, decreased performance status, and older age, as shown by a single institution retrospective analysis by Wasilewski et al. [51]. They reported that Grade 3 and 4 thromobocytopenia occurred two to three times more frequently than that reported by Perry et al., and that common side effects of fatigue and cytopenias were often severe enough to necessitate dose reduction, cessation of treatment, or additional medical intervention. Furthermore, they identified depression as another common comorbidity in elderly patients treated with RT and temozolomide, and highlighted polypharmacy and falls as negative prognostic indicators in this population.

On one hand, fit older patients with good performance status and favorable molecular subtypes may be undertreated with hypofractionated RT-based treatment [52]. However, on the other hand as Wasilewski et al., showed, an underestimation of toxicities in relatively robust clinical trial populations can misinform the risk/benefit ratio when making treatment decisions. Giaccherini et al. [53] performed a retrospective analysis in newly diagnosed GBM patients at least age 65 and treated with RT with or without concomitant temozolomide, that aimed to explore whether pre-treatment multidimensional assessment of frailty using validated tools could predict outcome. Using the Prognostic Nutritional Index (PNI), Charlson Comorbidity Index (CCI), Frailty Index (FI), along with other clinicopathologic factors, they confirmed previous findings suggesting high KPS and gross total resection were significantly associated with better OS. In addition, PNI, CCI, and FI were also all independent significant predictors of OS, thereby suggesting a role for validated measures of frailty in adjuvant treatment decision-making.

### 2.5. Considerations for Adjuvant Treatment

**Figure 1:**
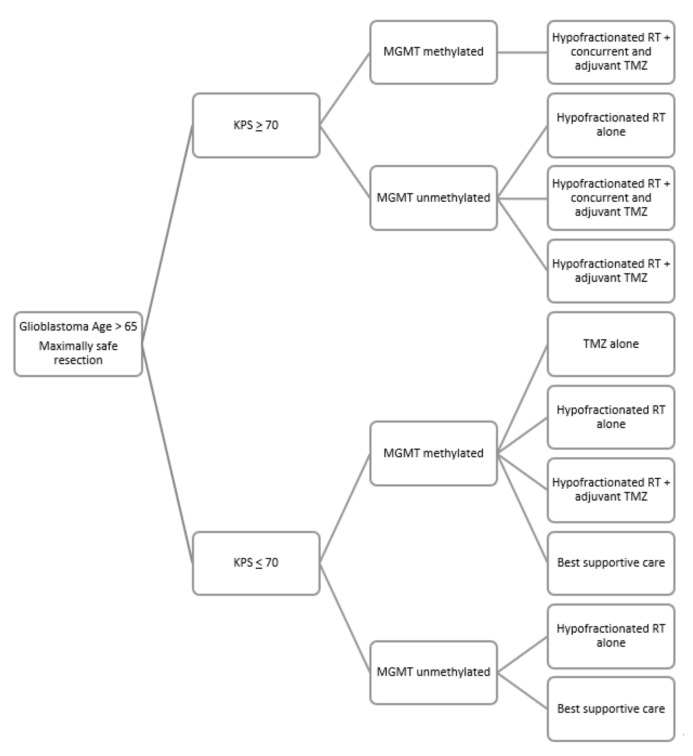
Proposed algorithm of post- operative management of elderly glioblastoma. Legend: KPS = Karnofsky Performance Status; MGMT = O6-methylguanine-DNA-methyltransferase; RT = radiotherapy; TMZ = temozolomide.

Consensus recommendations for treatment of older patients with GBM suggest that management should be based on the age, fitness of the patient, performance status, and MGMT promoter methylation status [54,55]. A general treatment algorithm for patients over age 65 with newly diagnosed GBM having undergone maximally safe surgical resection is provided in Figure 1.

The role of MGMT methylation status as a predictive marker of temozolomide response is well established in patients with glioblastoma of all ages [5,6,39]. However, only trials in the elderly have investigated different treatment allocations based on MGMT status [33,43] as an effort to limit toxicity from combined modality treatment. Therefore, since MGMT methylation status does not necessarily inform clinical decision-making in younger adults, its routine implementation in clinical practice has been challenging [56]. MGMT methylation testing is however recommended in elderly (over age 65–70) or frail patients as per the 2020 European Association for Neuro-Oncology (EANO) guidelines on the management of diffuse gliomas [10]. These guidelines along with other consensus recommendations such as those posed by the US-based National Comprehensive Cancer Network (NCCN) [57] recommend that elderly patients with MGMT methylation should have temozolomide incorporated as part of their regimen, whereas for those without methylation, especially those with poor performance status, treatment with hypofractionated RT alone should be considered. While consideration should be given to withholding temozolomide in this population, the results of the EORTC 26,062 study did suggest survival benefit with combined modality treatment in both the MGMT methylated and unmethylated groups. Therefore, discussion with the patient regarding a trial of temozolomide, especially with younger elderly patients (age 65–70) with good performance status, is warranted [56].

Performance status indeed plays a pivotal role in chemotherapy treatment recommendations regardless of MGMT status. Patients with good performance status (typically characterized by a KPS score of at least 60 or 70 or Eastern Cooperative Oncology Group Performance Status (ECOG-PS) score of 0–2) should be considered for combined modality treatment, whereas monotherapy is more highly recommended for patients with poor performance status [10,57]. Though both KPS and ECOG-PS scales are validated, they have limitations. Neither scale distinguishes between types of symptoms or performance limitations, and they are based on a clinician’s general impression of a patient’s symptoms and capacity to work [58,59]. Highlighting the potential error of such subjective evaluations, a previous study by Ando et al. [60] comparing ECOG-PS scores for patients with non-small cell lung cancer as recorded by oncologists, nurses, and the patients themselves showed significant differences between the groups. Oncologists gave the lowest (healthiest) scores, whereas patients reported the highest (poorest) scores. Moreover, the scales are neither sensitive nor specific, and are not able to adequately describe small changes in function or the true functional abilities GBM patients with diverse presentations and impairments depending on tumor location and extent [61]. For example, a patient with cognitive impairment may have the same KPS score as a patient with hemiparesis who is otherwise functional, despite having very different prognosis.

As such, older GBM patients represent a unique clinical cohort due to the complexity of distinguishing neuro-oncology related symptoms from pre-existing comorbidities and general fitness. In a cross-sectional survey of UK-based neuro-oncologists asking them to review their practice in assessing elderly GBM patients, Lorimer et al. [62] found that participants ranked performance status as the most important factor in determining treatment decisions. Only 18% of consultants routinely performed a cognitive or frailty screening test at consultation, but of those who performed those tests the results changed treatment decision approximately 50% of the time. Therefore, considering the heterogeneity of the elderly GBM population, performance status as determined by KPS or ECOG-PS is a crude measure of vulnerability within this cohort, and there is a clear need for specific geriatric assessment tools to help facilitate accurate and effective clinical decision-making.

## 3. Comprehensive Geriatric Assessment in Oncology

It is apparent that oncologic treatment regimens developed for and tested in adults are not necessarily suitable for older populations with variable physical and cognitive limitations, comorbidities, social situations, and organ function. Thus, the Comprehensive Geriatric Assessment (CGA) was introduced to oncology in the early 21st century and has since shown both prognostic and predictive value in cancer patients [14,63]. The CGA is designed to capture the functional age of older adults and identify those at increased risk of functional decline and reduced life expectancy. It is a multidimensional tool that uses validated metrics to evaluate age-related domains associated with morbidity and mortality in older adults [64,65]. Specifically, it assesses functional status and falls, cognitive function, comorbidities, polypharmacy, nutrition, psychologic state, socioeconomic issues, and geriatric syndromes [65].

The CGA has numerous benefits for oncologic care. As per Balducci’s criteria, the classical CGA classifies cancer patients in good general health as “fit”, patients with partial impairment in some domains as “vulnerable”, and patients with severe impairment in most domains as “frail”. These classifications have been demonstrated to have prognostic significance [66,67] and can be used for the purpose of determining rehabilitative potential, tolerance of treatment and stress, and selecting an appropriate treatment strategy [68]. It is therefore recommended by both the International Society of Geriatric Oncology (SIOG) [16] and the American Society of Clinical Oncology (ASCO) for use in patients age 65 or older who receive chemotherapy, with the goal of identifying vulnerabilities not detected during the typical oncologic assessment and developing an individualized treatment plan [17].

### A Role for the CGA in GBM

While the CGA has proven itself a useful tool for several different cancers, patients with CNS tumors had limited if any enrollment in the early pivotal trials of CGA implementation in oncology. Despite this, certain domains of the CGA have shown prognostic value for older adults with GBM. Of note, frailty (as described previously) [29], assessment of comorbidities, nutrition status, polypharmacy, cognitive deficit, and depression have all proven to be independent predictors of OS.

In a retrospective analysis of 34 patients with treated GBM age 65 or older, Giaccherini et al., evaluated the impact of clinical and biological factors including the Prognostic Nutritional Index (PNI) and Charlson Comorbidity Index (CCI) on outcomes. Both a CCI of three or more and a PNI below 42 demonstrated a clear but not statistically significant correlation with poorer OS [53]. Though CCI score did not reach statistical significance in this small cohort, a CCI score of greater than three was previously recognized to be significantly associated with poor OS in a retrospective review of 233 adult patients with GBM. It was further noted that patients over age 65 were significantly more likely to have a score greater than three [69]. Additionally, a high postoperative PNI was also associated with improved OS in a recent retrospective review of 335 GBM patients, though this investigation did not stratify for age [70].

Polypharmacy has also been attributed to reduced survival of malignant glioma patients over age 65, as Wasilewski et al., showed that patients prescribed eight or more medications had inferior OS compared to those prescribed less than eight medications [51]. In another retrospective study of 129 patients over age 65 with GBM who underwent total or subtotal resection, cognitive deficit was one of the preoperative factors that was independently associated with decreased survival [71]. A Mini Mental-State Examination (MMSE) score greater than 26 was also found to be associated with access to adjuvant therapy in an additional retrospective review, and this association correlated with improved survival in a cohort of GBM patients over age 70 [72]. Impairment in cognitive executive functioning was further shown to be independently associated with shortened survival in a general GBM population. The same analysis additionally showed that the presence of depressive symptoms as determined by the Beck Depression Inventory-II (BDI-II) was also associated with worse OS, though this was not specific to age [73]. An overview of the domains of the CGA, tools used to assess them, and their relevance in a GBM population is summarized in Table 2.

These previous studies all justify the rationale for inclusion of the CGA in the evaluation and management of elderly patients with GBM. However, due to the unique deficits caused by GBM, there is concern that geriatric assessment tools used in patients with extra-cranial malignancies may be less valid within a neuro-oncology cohort. For example, the measure of mobility in the modified G8 score is not necessarily appropriate for GBM management, and certain comorbidity assessments such as “history of heart failure or coronary artery disease” does not provide important information for GBM management [74]. Three studies have been published recently to address these concerns and determine whether the CGA and geriatric screening tools hold relevance in GBM.

The GOLDEN study by Lorimer et al. [75] is the first prospective trial investigating the use of the CGA in GBM. In this feasibility study, they developed a modified GA tool and assessed whether this could be implemented within busy outpatient clinics. The modified CGA focused on neurological symptoms but used previously validated questionnaires: Lawton and Brody Instrumental Activities of Daily Living (IADLs) [76], the Hospital Anxiety and Depression Scale (HADS) [77], the Geriatric 8 screening questionnaire (G8) [78], the Montreal Cognitive Assessment tool (MoCA) [79], the Trail Making Test B (TMTB) [80], ECOG-PS, and CCI [81]. Fifty patients over age 65 with GBM presenting for discussion of post-surgical treatment options were recruited, at a rate of 82%. More than 85% of patients felt they had enough time to understand the study and complete the questionnaires, and 81% reported that they would participate in a similar study again. Completeness rate for all GA questionnaires exceeded 80% (except TMTB, which was 70%). Three factors were associated with survival on multivariate analysis: radical treatment, abnormal MoCA score, and mobility impairment, though the study was not powered to detect statistical significance. Staff members reported that though assessment required more time in clinic, they considered it a worthwhile endeavor given the information obtained, such as objective measurements that empowered their treatment decision-making. This study therefore highlights that the adoption of a CGA tool into neuro-oncology practice is feasible and acceptable to patients and staff.

Given concern that geriatric screening tools such as the G8 may not be relevant for the management of GBM, Deluche et al. [74] performed a retrospective analysis of GBM patients between age 65 and 89 to establish whether the use of the G8 before adjuvant treatment is appropriate in this cohort. Patients were classified into three groups based on their score to identify those with poor prognosis: a high score group (G8 score 14.4 to 17), intermediate score group (score 10.5 to 14), and low score group (score < 10.5). Patients in the high score group were more likely to receive combined chemoradiation, with only one patient in this cohort not receiving combined modality treatment. A total of 92% of patients with an intermediate score received combined chemoradiation, and amongst those with a low score 44% were treated with chemoradiation and 27% received palliative care. Median OS was 4 months in the low score group, 15 months in the intermediate score group, and 42 months in the high score group. Age was associated with G8 score category, and after multivariate analysis, the absence of RT and being in the low G8 score group were both independent predictors of poorer OS. The authors concluded that the G8 provided valuable information to better identify patients with poor prognosis, that the G8 screening tool enabled the collection of a wide range of relevant information, and that it was suitable for GBM patients.

Lombardi et al. [82] additionally reported a retrospective analysis of a single-center study assessing the predictive role of the CGA in patients aged 65 to 84 years with newly diagnosed GBM. The metrics evaluated in this CGA were the Cumulative Illness Rating Scale-Comorbidity (CIRS-CI) and Severity Index (CIRS-SI) [83], Activities of Daily Living (ADL) [84], Instrumental Activities of Daily Living (IADL) [84], the MMSE [85], and the Geriatric Depression Scale (GDS) [86]. Patients were stratified into fit, vulnerable, and frail categories based on the CGA results. Combined chemoradiotherapy was administered to 98% of fit patients, 90% of vulnerable patients, and 52% of frail patients. Of note, frail patients received fewer cycles of maintenance temozolomide compared to vulnerable and fit patients (2.8 vs. 5 vs. 5.2 cycles, respectively). Patients with a KPS between 40 and 60% usually received monotherapy with temozolomide or radiation depending on MGMT methylation status. Median survival was 10.3 months in the frail group,12.1 months in the vulnerable group, and 16.5 months in the fit group. On multivariate analysis the CGA score was an independent predictor of survival, with frail patients having significantly reduced OS relative to the vulnerable and fit patient groups. Notably, although there was an association between KPS and CGA, 47% of patients deemed “frail” with the CGA had a KPS between 70 and 100 and were therefore treated with combined modality chemoradiation, raising concern amongst the authors that they may have been overtreated. This is in agreement with previous data showing that in a general oncology population, though performance status was associated with some measures of the CGA, several aspects of functional impairment were not, such that between 9–37% of patients with good ECOG-PS (<2) had additional limitations detected by CGA [13]. Overall, this study showed that the CGA was a significant predictor of survival in elderly GBM, and may be a useful treatment decision tool.

## 4. Conclusions and Future Directions

Management of GBM in older adults has been a major topic of review within the last few years, suggesting that there are still questions regarding how to choose appropriate treatment for such a heterogenous population. The main predictors of survival are age, performance status, and MGMT methylation status, and it is upon these factors that the majority of current treatment algorithms are based. MGMT status has a clear and definitive predictive and prognostic role, and guiding management based on this molecular marker is effective and appropriate. On the other hand, chronologic age provides less of a clear algorithmic delineation as the health status of the older adults varies considerably. Certain measures of biological age such as sarcopenia are being investigated as prognostic measures for glioblastoma, and preliminary investigations have suggested correlation with KPS in patients with progressive disease [87]. As discussed however, currently used performance status metrics of KPS and ECOG-PS are crude measures of fitness and are not necessarily appropriate for GBM patients. The CGA is by definition a more comprehensive evaluation of the health status of elderly patients, and has predictive and prognostic relevance in numerous cancers. Its potential use in GBM is now starting to be explored.

Thus far, studies of the CGA in GBM have focused on using geriatric assessment tools prior to administration of adjuvant chemotherapy and hypofractionated radiation. However, the CGA may be useful for surgical risk assessment as well, as the previous study by Cloney et al. [29] suggested that frailer patients were less likely to undergo maximal surgical resection and had poorer outcomes. Additionally, though hypofractionated radiation is considered standard of care for older GBM patients, there remains equipoise over whether patients aged 65 to 70 may benefit from standard radiation, given that the trial by Stupp et al. [5] included patients up to age 70. In this case the CGA may be of benefit for choosing the most suitable treatment for these patients. Furthermore, given that long-term survival is limited in GBM patients, especially in older populations, appropriate palliative care is an important facet of management. The CGA has demonstrated the ability to identify previously unknown geriatric conditions that can contribute to potential toxicity and treatment discontinuation, and therefore, may have a role in guiding palliative care measures that can improve quality of life.

The recent focus on management of older GBM patients is necessary given the aging population and high morbidity and mortality associated with this disease. Though questions remain regarding optimal testing in a neuro-oncology population and practicality of administration, recent studies utilizing the CGA in this cohort have laid an important foundation on which future prospective trials can further establish a role for the CGA in in guiding treatment decisions.

## Figures and Tables

**Table 1:** Pivotal trials providing evidence for adjuvant therapy in older adults with GBM.

Adjuvant Treatment	Trial	Age Cut-Off	Treatment Intervention	Treatment Control	Outcome
Radiotherapy	Roa et al., 2004 [32]	>60 years	40 Gy RT in 15 fractions (3 weeks)	60 Gy RT in 30 fractions (6 weeks)	OS 5.6 vs. 5.1 months (*p* = 0.57)
	Malmstrom et al., 2012 [33]	>60 years	34 Gy RT in 10 fractions (2 weeks)	60 Gy RT in 30 fractions (6 weeks)	OS 7.5 vs. 6.0 months (HR 0.85, *p* = 0.24)Age > 70: HR 0.59, *p* = 0.02
	Roa et al., 2015 [34]	Frail = age ≥; 50 years and KPS 50–70 Elderly and frail = age ≥ 65 and KPS 50–70 Elderly = age ≥; 65 and KPS 80–100	25 Gy RT in 5 fractions (1 week)	40 Gy RT in 15 fractions (3 weeks)	OS 7.9 vs. 6.4 months (*p* = 0.988)
Chemotherapy	Malmstrom et al., 2012 [33]	>60 years	TMZ (200 mg/m^2^ for 5 days Q28 days, up to 6 cycles)	60 Gy RT in 30 fractions (6 weeks) 34 Gy RT in 10 fractions (2 weeks)	OS 8.3 vs. 6.0 months (HR 0.70, *p* = 0.01)Age > 70: HR 0.35, *p* < 0.0001OS 8.4 vs. 7.4 months (HR 0.82, *p* = 0.12) OS MGMT methylated vs. umethyalted: 9.7 vs. 6.8 months (HR 0.56, *p* = 0.02)
	Wick et al., 2012 [43,44]	>65 years	TMZ (100 mg/m^2^ 1 week on, 1 week off)	60 Gy RT in 30 fractions (6 weeks)	OS 8.6 vs. 9.6 months (HR 1.15, *p*_non-inferiority_ = 0.033) OS MGMT methylated vs. umethyalted: 11.9 vs. 8.2 months (HR 0.62, *p* = 0.014) MGMT methylated, TMZ vs. RT: 18.4 vs. 9.6 months (HR 0.44, *p* < 0.001)
Combined Chemoradiotherapy	Perry et al., 2017 [6]	≥65 years	40 Gy RT in 30 fractions (3 weeks) with concurrent TMZ (75 mg/m^2^ daily) + adjuvant TMZ (150–200 mg/m^2^ for 5 days Q28 days up to 12 cycles)	40 Gy RT in 30 fractions (3 weeks)	OS 9.3 vs. 7.6 months (HR 0.67, *p* < 0.001) OS MGMT methylated: 13.5 vs. 7.7 months (HR 0.53, *p* > 0.001) OS MGMT unmethylated: 10.0 vs. 7.9 months (HR 0.75, *p* = 0.055)

Legend: RT = radiotherapy; TMZ = temozolomide; OS = overall survival; HR = hazard ratio; MGMT = O^6^-methylguanine-DNA-methyltransferase.

**Table 2:** Commonly assessed domains in the Comprehensive Geriatric Assessment.

Assessment Domain	Commonly Used Tools	Rationale for Use	Evidence in GBM Outcomes
Functional Status/Frailty	ADLs, IADLs	Improved overall survival associated with independence in IADLs Impaired function associated with increased risk of toxicity due to chemotherapy	Yes
Comorbidities	CCI	Comorbidity is increased with poorer survival, chemotherapy toxicity, and hospitalizations	Yes
Cognitive Function	Mini-Cog, MMSE, MoCA	Patients with cancer and cognitive deficit have worse survival than those with normal cognitive function Impairment may impact adherence to treatment, understand follow-up instructions, and increase risk of cognitive side effects of treatment	Yes
Nutrition	PNI, mini nutritional assessment	Malnutrition impacts drug metabolism, functional status, falls risk Weight loss is independent prognostic factor survival and is associated with lower poorer performance status in cancer patients	Yes
Polypharmacy	Medication review	Cancer-related therapy increases risk for adverse effects, interactions, and nonadherence due to regimen complexity	Yes
Psychological State	Geriatric depression scale, BDI-II	Prevalence of clinically significant depression in up to 25% of older patients with cancer Associated with increased risk of functional decline and utilization of healthcare resources	Yes
Social Support	Medical outcomes study social support	Those with poor social support at highest risk of depression Elderly patients often require assistance from caregivers to successfully complete treatment; those with poor social networks more likely to have worse outcomes	Unclear

Legend: ADLs = Activities of Daily Living; IADLs = Instrumental Activities of Daily Living; CCI = Charlson Comorbidity Index; MMSE = Mini-Mental State Examination; MoCA = Montreal Cognitive Assessment; PNI = Prognostic Nutritional Index; BDI-II = Beck Depression Inventory-II.
